# Validation of Dietary Antioxidant Index (DAI) and investigating the relationship between DAI and the odds of gastric cancer

**DOI:** 10.1186/s12986-020-00529-w

**Published:** 2020-12-01

**Authors:** Farhad Vahid, Diana Rahmani, Seyed Hossein Davoodi

**Affiliations:** 1 Department of Nutritional Sciences, Arak University of Medical Science, Arāk, Iran; 2 Department of Nutritional Sciences, Faculty of Nutrition Sciences and Food Technology, National Nutrition and Food Technology Research Institute, Shahid Beheshti University of Medical Sciences, Tehran, Iran; 3 Cancer Research Center, Shahid Beheshti University of Medical Sciences, Tehran, Iran

**Keywords:** Gastric cancer, Dietary Antioxidant Index (DAI), Total antioxidant capacity (TAC), Malondialdehyde (MDA), Food Frequency Questionnaire (FFQ)

## Abstract

**Background:**

Gastric cancer (GC) incidence and mortality are rapidly growing worldwide. It is estimated that more than 1,000,000 new cases are diagnosed each year, and more than 78,000 people lose their lives due to GC. The association between dietary antioxidants and GC has been shown in some studies. However, because of the discrepancy between the findings and the lack of a valid indicator, it seems necessary to design and validate the Dietary Antioxidant Index (DAI) to examine the diet's total antioxidant content. The present study aimed to survey the validity of DAI and its association with the odds of GC.

**Methods:**

In this hospital-based case–control study, 82 patients with GC and 95 healthy controls were examined. We used a 168-item food frequency questioner to assess dietary intakes. The DAI was calculated based on the intake of vitamin A, C, E, and selenium, manganese, and zinc. We standardized each of the six vitamins and minerals by subtracting the global mean and dividing by the global standard deviation to calculate DAI. We then calculated the DAI by summing up the standardized intakes of these vitamins and minerals of the individuals with equal weight.

**Results:**

We observed a significant correlation between DAI and total antioxidant capacity (TAC) after controlling for age, body mass index (BMI), energy intake, smoking and fasting blood sugar, education, total fat intake, helicobacter pylori infection, total cholesterol, and saturated fatty acid (SFA) intakes. Results obtained from modeling DAI as a continuous variable in relation to GC showed a negative association after adjustment for age and in the multivariable analysis (*OR* = 0.64, *CI* = 0.43–0.95).

**Conclusion:**

DAI is a valid indicator of dietary antioxidants assessments, and it can be used as a predictor of antioxidant status due to its correlation with serum antioxidant levels. The results showed that dietary antioxidants have a significant relationship with GC, which indicates the importance of antioxidants in this cancer's etiology.

## Introduction

Gastric Cancer (GC) is one of the most common and pernicious malignancies globally [1]. It is estimated that each year more than 1,033,701 new cases of GC are diagnosed, and more than 782,685 people lose their lives due to GC [2]. GC's lifetime risk to age 74 remains between 5 and 20% in some parts of Asia [2, 3]. Given that the therapies for GC are limited, and the survival chance is low, prevention can be a very effective strategy to reduce the mortality from GC [4–6]. Several studies have found a link between the incidence of GC and unhealthy diets [7, 8]. In addition, the association between dietary antioxidants and the reduction of the incidence and prevalence of GC has been observed [9–11]. Therefore, the diet's total antioxidant content can provide a comprehensive overview of the relationship between dietary antioxidants and the incidence and/or risk of GC [12]. Several studies have shown that some foods and micronutrients have antioxidant or/and anti-inflammatory properties [7, 13–16]. Specifically, due to the increase in the incidence and prevalence of various types of cancers, some foods and micronutrient antioxidants are considered more. [18, 20]. Several studies have examined the association between food and micronutrients with antioxidant properties with the incidence and prevalence of various diseases [17–19]. In addition, natural products play a critical role in discovering and developing numerous drugs for the treatment of various types of cancers via different mechanisms [21–23]. These studies have also done well in cancer patients [18, 24–26].

However, most studies have investigated the relationship between foods or micronutrients with antioxidant properties individually or in a limited way, considering the whole diet. Dietary antioxidants can affect interdependent effects and may have a different total effect. Thus, Dietary Antioxidant Index (DAI) was designed to examine the diet's entire antioxidant content. This index has been used in some studies, and significant results have been observed [27–29]. The DAI posits that people's diets can be divided into two major categories: mainly anti-oxidative or mainly oxidative [27]. While further investigation and validation are needed to determine the 'DAI's sensitivity and specificity [27], it can be used to study the nutritional status in a wide range of outcomes. Therefore, this study's main goals are to investigate 'DAI's correlation with the antioxidant level in blood and survey its validity.

Differences in GC subgroups and their relationship with the DAI can respond to how the antioxidant system might influence the mechanisms of prevention or treatment of this cancer [9, 30, 31]. While the possible mechanisms of how dietary antioxidants can prevent or even, in some cases, eliminate cancer cells are very limited, there are several suggested hypotheses for these mechanisms. Antioxidants may inhibit or limit the formation of potential carcinogens (e.g., N-nitroso compounds) associated with GC [9]. A diet high in lipids causes oxidative and inflammatory stress, mediated by cytokines such as tumor necrosis factor-a (TNF-a) and interleukin (IL)-6 and oxidized lipids. The presence of antioxidants-rich foods during a high-fat diet might provide a depot of extrinsic antioxidants, slake radical species produced at the gastric level, and synergize with esoteric antioxidants and providing more efficient protection against oxidative stress [32].

Therefore, considering the limited resources and studies on validating the DAI, the present study examined this index's validity and its association with GC's odds.

## Method and materials

### Participant

The full protocol of this study has already been published elsewhere [8]. In summary, this hospital-based case–control study was conducted from December 2014 to May 2016. Eighty-two patients with GC and ninety-five healthy controls were examined. The cases were patients with GC who were diagnosed by a gastroenterologist within the previous month. Controls were randomly selected from among other 'patients' caregivers attending the same clinics. Controls were frequency-matched on sex and age (± 5 years). Data on cases and controls were collected simultaneously, and both groups were interviewed in the same setting. Informed consent was received from all participants. The local Ethics Review Committee approved the study protocol at Shahid Beheshti University of Medical Sciences, Tehran, Iran.

### Inclusion and exclusion criteria

#### Inclusion criteria

(a) in the control group: the absence of malignancy, pregnancy, lactation or a history of cancer, neurological, gastrointestinal, hepatic, endocrine, immune, kidney and heart disorders and diseases, (b) in case and control groups: the absence of special diets such as vegetarian, or the diets resulting in weight reduction or increase during the year before the interview, (c) in case group: the absence of conditions such as pregnancy, lactation, neurological, gastrointestinal, hepatic, endocrine, immune, kidney and heart disorders and diseases, (d) in both group: being in the age range of 20–80 years and (e) willingness to cooperate in the study.

#### Exclusion criteria

(a) To the lack of adherence to the study protocol, (b) major diet changes during the study, including diets aimed at weight increase or decrease, (c) reported intake energy over 5500 or less than 800 kcal/day.

### Assessment of antioxidants markers and blood samples

#### Participant preparation

The case and controls should not have been on any corticosteroids, anti-inflammatory medications, or painkillers for at least 48 h before collecting specimens.

#### Biofluid analysis

After fasting for 10–12 h, venous blood samples (10 ml) were taken in vacutainer tubes under sterile conditions from participants between 08:30–10:30 am. Serum was obtained from freshly drawn, rapidly centrifuged. The serum was quickly frozen at − 70 °C and stored until processed [8].

The serum levels of antioxidants markers, including total antioxidant capacity (TAC) and Malondialdehyde (MDA) for all participants, were measured using Ferric-reducing antioxidant power (FRAP) and Thiobarbituric acid assay (TBA) methods. All participants' antioxidant levels were measured using kits/martials materials produced by Pishtaz Teb Zaman Diagnostics Co., Ltd, and Shanghai Crystal Day Biotech Co., Ltd provided by Negin Salamat Saba Co. Also, serum levels of the low-density lipoprotein (LDL), high-density lipoprotein (HDL), and triglyceride were extracted from patients' medical records.

### Assessment of dietary intake

We used a 168-item food frequency questionnaire (FFQ) to assess the case's dietary intakes and controls over the past year. Case and controls were asked to report each food item's frequency of consumption in the last year according to the standard size units (standard serving size) in the questionnaire.

The information obtained from the questionnaires was then analyzed using Nutritionist IV (First Databank, Hearst Corp., SanBruno, CA, USA) to calculate the average daily intake of energy and nutrients. To calculate the DAI, we use the daily intake of food items affecting the index of antioxidants.

### Calculation of DAI and DAQS Scores

We used two previously validated methods to derive dietary antioxidant indices. The first method, the Dietary Antioxidant Quality Score (DAQS), was adapted from Rivas et al. [33]. The DAQS is calculated based on the intake of six antioxidant vitamins and minerals (vitamin A, vitamin C, vitamin E, selenium, manganese, and zinc) derived from the FFQ. The obtained DAQS was used to calculate antioxidant-nutrient intake. The score refers to the intake of specific vitamins and minerals that have been proven to act as dietary antioxidants: selenium, zinc, vitamin A, vitamin C, and vitamin E. Daily nutrient intake was compared to that of the Recommended Daily Intake (RDI). The intake of each of the five evaluated antioxidant nutrients was assessed separately by assigning a value of zero or one to each nutrient. When the intake was below 2/3 of the RDI, it was assigned a value of zero. Similarly, when the intake was higher than 2/3 of the RDI, it was assigned a value of 1. Thus, the DAQS ranged from 0 (pro-oxidative diet) to 5 (anti-oxidative diet).

The second method, the DAI, was developed by Wright et al. [27]. We standardized each of the same six dietary vitamins and minerals by subtracting the global mean and dividing the result by the global standard deviation to estimate DAI. We then calculated the DAI by summing up the standardized intakes of these vitamins and minerals and equal weight, as described next [27, 28, 33]:

$${\text{DAI}} = \mathop \sum \limits_{{{\text{i}} = 1}}^{{{\text{n}} = 6}} \frac{{{\text{Individual}}\;{\text{Intake}} - {\text{Mean}}}}{{{\text{SD}}}}$$

### Assessment of other variables

For all participants, the required information about age, sex, place of birth (rural/urban), smoking, alcohol consumption, aspirin/nonsteroidal anti-inflammatory drug (NSAID) use, regular physical activity, education, and family history of cancer was collected through a general information questionnaire during the interviews.

The weights of participants were measured with the least clothes using a SECA digital scale. The height was measured without shoes in standing position, leaning against the wall and shoulder blades under normal circumstances using SECA 206 body meter (Wall mounted height measuring tape). Body Mass Index (BMI) was calculated by dividing weight (in kilograms) by the square of height (square meters).

### Statistical analyses

Descriptive analyses were carried out using paired t-test for the continuous variables and Chi-square test for the categorical variables. DAQS (as dichotomous) was examined across the following characteristics: age, sex, BMI, education, smoking, alcohol, *H*. *pylori* infection, physical activity, aspirin/NSAID use, and family history of cancer. Analyses focusing on the association of DAI scores and antioxidant markers were carried out using DAI as a continuous variable. For analyses focusing on GC as an outcome, the DAI was analyzed both as a continuous variable and as a dichotomous variable, categorized based on the median value of the DAI for the controls (− 0.19). Beta estimates and 95% confidence intervals (CI) for the antioxidant markers were estimated using linear regression and odds ratios (OR), and 95% CI for GC as an outcome was value assessed using logistic regression models, adjusting only for age and then fitting a model with additional adjustment for alcohol consumption, marital status, physical activity, cancer history in the first-degree family, total antioxidant capacity, vitamin E, manganese, and salt intake. The partial correlation was used to estimate the coefficients between DAI and serum levels of antioxidant factors in the subjects. Statistical tests were performed using SPSS 21; all *p* values were based on two-sided tests.

## Results

Table 1 shows the distribution of 82 cases of GC and 95 controls according to the selected variable [7, 8]. The mean age was 48.33 ± 10.74 and 51.36 ± 11.81 in the case and the control groups, respectively. Controls were significantly had higher BMI and DAI scores compared to controls. The mean DAI value for controls was 0.17 (SD = 1.18) and for the cases were − 0.21 (SD = 0.68), indicating a more antioxidant diet for controls (p value = < 0.001). The distribution of characteristics and dietary intakes across DAQS categories were shown in Table 2. Control characteristics by DAI categories are provided in Table 3.

**Table 1:** Distribution of 82 gastric cancer cases and 95 controls according to selected variables^a, b^ [8, 38]

Characteristics	Mean ± SD or N (%)		p value
	Controls (n = 95)	Cases (n = 82)	
Age (years)	48.33 ± 10.74	51.36 ± 11.81	0.07
Body Mass Index (BMI, kg/m^2^)	24.96 ± 2.71	26.36 ± 5.12	0.02
Dietary antioxidant Index (DAI)	0.17 ± 1.18	− 0.21 ± 0.68	< 0.01
TAC (mmol/l)	1.91 ± 1.27	1.16 ± 1.26	< 0.01
MDA (μmol/l)	3.01 ± 1.68	3.72 ± 2.19	< 0.01
Sex			0.98
Females	52 (54.74)	45 (54.88)	
Males	43 (45.26)	37 (45.12)	
Education			0.24
Diploma or less	67 (70.53)	51 (62.20)	
Higher than diploma	28 (29.47)	31 (37.80)	
Smoking			0.82
Never smoker	80 (84.21)	68 (82.93)	
Ever smoker	15 (15.79)	14 (17.07)	
Alcohol			0.41
Non drinker	86 (90.53)	71 (86.59)	
Drinker	9 (9.47)	11 (13.41)	
*H. pylori* infection			< 0.01
Negative	46 (48.42)	21 (25.61)	
Positive	49 (51.57)	61 (74.39)	
Regular physical activity			0.03
Yes	30 (31.58)	14 (17.07)	
No	65 (68.42)	68 (82.93)	
Aspirin/NSAID use			0.83
No	86 (90.53)	75 (91.46)	
Yes	9 (9.47)	7 (8.54)	
Cancer history			0.41
Yes	11 (11.58)	13 (15.85)	
No	84 (88.42)	69 (84.15)	

*TAC* total antioxidant capacity, *mmol/l* millimoles per liter, *MDA* malondialdehyde, *μmol/l* micromoles per liter

^a^Comparison of mean of case and control groups in case of a normal distribution of variables by the t-test and abnormal distribution of variables by the Mann–Whitney test

^b^Chi-square was used for categorical variables

**Table2:** Distribution of characteristics and dietary intakes across dietary antioxidant quality score (DAQS) categories^a, b^ (n = 177)

Characteristics	Mean ± SD		*p* value
	DAQS ≤ 3 (n = 88)	DAQS > 3 (n = 89)	
Age (years)	49.59 ± 11.30	50.07 ± 11.45	0.79
Weight (kg)	59.93 ± 7.47	59.10 ± 7.36	0.48
Height (cm)	165.86 ± 8.31	167.74 ± 7.70	0.15
BMI (k/m^2^)	25.95 ± 4.21	24.85 ± 3.62	0.09
TNF-a (pg/ml)	30.74 ± 26.09	27.51 ± 24.88	0.44
TG (mg/dl)	139.40 ± 57.50	136.24 ± 58.63	0.73
HDL (mg/dl)	46.66 ± 10.60	47.91 ± 12.37	0.49
LDL (mg/dl)	100.05 ± 30.18	104.98 ± 31.35	0.32
Energy intake (kcal/day)	3006.24 ± 592.47	2990.36 ± 570.30	0.86
Protein (gr/day)	106.21 ± 37.52	104.24 ± 44.34	0.76
Carbohydrate (gr/day)	378.92 ± 122.43	371.84 ± 101.38	0.70
Total Fat Intake (gr/day)	111.97 ± 39.12	114.25 ± 36.30	0.71
Saturated fatty acid (gr/day)	45.56 ± 33.90	47.56 ± 31.61	0.71
Monounsaturated fat (gr/day)	29.31 ± 10.91	30.95 ± 9.44	0.33
PUFA (gr/day)	30.49 ± 19.01	28.49 ± 18.64	0.51
MUFA 18 1 (gr/day)	23.25 ± 10.07	24.76 ± 9.40	0.34
PUFA 18 2 (gr/day)	25.00 ± 18.30	22.85 ± 18.05	0.47
PUFA 18 3 (gr/day)	3.70 ± 2.25	4.14 ± 2.00	0.22
Sodium (mg/day)	4109.37 ± 1664.42	3723.29 ± 1381.93	0.11
Potassium (mg/day)	3910.73 ± 1306.06	3692.42 ± 1276.97	0.30
Vitamin A (Microgram/day)	565.66 ± 243.72	821.25 ± 375.33	< 0.01
Beta-carotene(Microgram/day)	5250.19 ± 2197.20	5152.00 ± 2145.91	0.78
Alfa-carotene (Microgram/day)	818.54 ± 446.58	784.44 ± 390.25	0.62
Lutein (Microgram/day)	2241.78 ± 1006.68	2566.62 ± 1459.05	0.08
Beta-cryptox (Microgram/day)	338.73 ± 160.31	320.51 ± 172.22	0.49
Lycopene (Microgram/day)	5047.17 ± 2329.06	5333.80 ± 2126.44	0.43
Vitamin C (mg/day)	149.66 ± 62.20	175.31 ± 70.63	< 0.01
Calcium (mg/day)	1225.30 ± 466.16	1162.47 ± 347.51	0.37
Iron (mg/day)	18.62 ± 5.87	19.80 ± 5.18	0.18
Vitamin D (Microgram/day)	2.14 ± 1.59	2.30 ± 1.66	0.56
Vitamin E (mg/day)	16.69 ± 6.61	23.89 ± 8.41	< 0.01
Alfa-tocopherol (mg/day)	7.47 ± 4.70	11.36 ± 5.01	< 0.01
Thiamin (mg/day)	2.20 ± 0.93	2.14 ± 0.80	0.70
Riboflavin (mg/day)	2.12 ± 0.72	2.24 ± 0.79	0.33
Niacin (mg/day)	28.40 ± 9.76	31.65 ± 11.51	0.05
Vitamin B6 (mg/day)	2.41 ± 0.93	2.51 ± 0.94	0.48
Folate (Microgram/day)	692.70 ± 236.78	679.14 ± 238.61	0.72
Vitamin B12 (Microgram/day)	5.54 ± 3.50	5.47 ± 2.78	0.89
Biotin (Microgram/day)	38.42 ± 12.82	37.65 ± 14.59	0.72
Pantothenic (mg/day)	7.25 ± 2.57	8.24 ± 2.68	0.02
Vitamin K (Microgram/day)	297.55 ± 152.78	255.96 ± 142.10	0.08

*kg* kilogram, *cm* centimeter, *BMI* Body Mass Index, *k/m*^*2*^ kilogram per square meter, *TNF-a* Tumor necrosis factor alpha, *pg/ml* pictogram per milliliter, *mg/dl* milligram per deciliter, *TG* triglyceride, *HDL* high-density lipoprotein, *LDL* low-density lipoprotein, *gr* gram, *Mufa* Monounsaturated fat, *Pufa* Polyunsaturated fatty acid, *mg* milligram, *gr* gram

^a^Comparison of mean of case and control groups in case of a normal distribution of variables by the t-test and abnormal distribution of variables by the Mann–Whitney test

^b^Chi-square was used for categorical variables

**Table 3:** Participant characteristics by level of the dietary antioxidant index (DIA) among controls, Iranian Gastric Cancer case–control study^a, b^ (n = 95)

Characteristics	Mean ± SD or N (%)		*p* value
	DAI ≤ 0.41 (n = 48)	DAI > 0.41 (n = 47)	
Age (years)	48.31 ± 11.52	48.36 ± 10.01	0.98
Sex			0.91
Female	26 (54.16)	26 (55.31)	
Male	22 (45.83)	21 (44.69)	
Body Mass Index (kg/m^2^)	25.36 ± 2.73	24.55 ± 2.65	0.14
Family history of cancer			0.72
Yes	5 (10.41)	6 (12.76)	
No	43 (89.58)	41 (87.23)	
Education			0.33
Less than a high school and diploma	36 (75)	31 (65.95)	
Higher than diploma	12 (25)	16 (34.04)	
Smoking			0.81
Yes	8 (16.66)	7 (14.89)	
No	40 (83.33)	40 (85.10)	
Alcohol			0.27
No	45 (93.75)	41 (87.23)	
Yes	3 (6.25)	6 (12.76)	
Regular Physical Activity			0.60
Yes	14 (29.16)	16 (34.04)	
No	34 (70.83)	31 (65.95)	
*H. pylori* infection			0.47
Yes	23 (47.91)	26 (55.31)	
No	25 (52.08)	21 (44.68)	
Aspirin/NSAID use			0.70
No	44 (91.66)	42 (89.36)	
Yes	4 (8.33)	5 (10.63)	

^a^Comparison of mean of case and control groups in case of a normal distribution of variables by the t-test and abnormal distribution of variables by the Mann–Whitney test

^b^Chi-square was used for categorical variables

Partial correlation was observed between DAI and serum levels of antioxidant factors in the subjects (Table 4). In model 1, controlling for age, BMI, energy intake, and smoking, acceptable and significant correlation were seen between the DAI and TAC. However, the results did not show a significant correlation between MDA and serum levels of antioxidant factors. In model 2, after multivariable adjustments, the TAC results were improved, and the correlation observed was stronger and more significant. However, there was still no significant correlation for MDA.

**Table 4:** Partial correlation coefficients between dietary antioxidant index (DIA) and serum levels of antioxidant factors (n = 177)

Model 1	Correlation coefficient	*p* value^a^	Model 2	Correlation coefficient	*p* value^b^
TAC (mmol/l)	0.25	0.04	TAC	0.41	< 0.01
MDA (μmol/l)	0.10	0.41	MDA	0.09	0.47

*TAC* total antioxidant capacity, *mmol/l* millimoles per liter, *MDA* malondialdehyde, *BMI* Body Mass Index, *μmol/l* micromole per liter, *μmol/l* micromoles per liter

^a^Controlling for age, BMI, energy intake, and smoking

^b^Additionally controlling for fasting blood sugar, education, total fat intake, *H. pylori* infection, total cholesterol, and saturated fatty acid intakes

Beta estimates and 95% confidence intervals for DAI and antioxidant markers are shown in Table 5. significant and acceptable correlation between DAI and TAC were observed after controlling for age, BMI, energy intake, and smoking (model 1), and Additionally controlling for fasting blood sugar, education, total fat intake, H. pylori infection, total cholesterol, and saturated fatty acid intakes (model 2). Nevertheless, there the correlation between DAI and MDA was non-significant and unacceptable in both models.

**Table 5:** Odds ratios and confidence intervals for the association between dietary antioxidant index (DIA) and gastric cancer (n = 177)

DAI	DAI (categorical)^c^ OR and 95% CI		*p* value ^a^	DAI (continuous) OR and 95% CI	*p* value
	− 0.19 ≤	− 0.19 >			
	1 (ref.)	0.68 (0.37–1.24)	0.20^a^	0.65 (0.45–0.93)	0.02^a^
	1 (ref.)	0.72 (0.36–1.42)	0.34^b^	0.64 (0.43–0.95)	0.02^b^
DAQS	DAQS (categorical) OR and 95% CI				
	DAQS ≤ 3	DAQS > 3			
	1 (ref.)	1.32 (0.69–2.53)	0.39^a^		
	1 (ref.)	1.30 (0.61–2.73)	0.48^b^		

^a^Age-adjusted

^b^ Additionally adjusted for gender, body mass index, smoking, education, *H. pylori* infection, alcohol consumption, aspirin/NSAIDs use, physical activity, cancer history in the first-degree family, total energy intake

^c^Median

ORs and 95% CIs for the odds of GC according to dichotomized DAI scores are shown in Table 5. The results obtained from modeling DAI as a continuous variable in relation to odds of GC showed a negative association after adjustment for age (OR = 0.65; 95% CI = 0.45–0.93) and in the multivariable analysis (OR = 0.64, CI = 0.43–0.95). When the analysis was carried out with DAI expressed as a dichotomous variable and adjusting for age, subjects with DAI score − 0.19 ≤ were at higher odds of having GC compared to subjects with DAI − 0.19 > . However, the OR was not significant (OR_DAI_ > − 0.19 / ≤ − 0.19 = 0.68; 95% CI = 0.37–1.24). In addition, after multivariable adjustment, a higher non-significant odd of having GC were seen. No significant association was seen after analyzing using DAQS and adjusting for age and multivariable adjustment.

## Discussion

The results showed that there is an acceptable and significant correlation between DAI and TAC. Validation of DAI allows researchers to use this proprietary index to examine a comprehensive aspect of the diet in nutritional assessments and researches. There is limited research on the relationship between the total intake of antioxidants and their serum changes. Most studies on dietary antioxidants and their effect(s) on the serum antioxidant levels have examined one or two antioxidants. The interdependent impact of different antioxidants can affect overall antioxidant levels of serum. Therefore, getting total antioxidants intake from the diet in a coherent formula and designing an index to predict serum levels of antioxidants is an effective step in assessing nutritional status. The correlations observed in this study showed that this indicator is used in different studies and different outcomes. The correlation between DAI and the TAC was significant and acceptable, while DAI's correlation with MAD was not significant. One explanation could be that TAC is more susceptible to dietary changes, and it is expected that TAC levels modify according to dietary antioxidant intake. Previously, Wang et al., in 2012, concluded that TAC is a good predictor of dietary and plasma antioxidant status" [34]. These results were in line with the current findings and the significant partial correlation between the DAI and the TAC after multiple controlling for confounders.

In addition, our study showed that DAI is associated with odds of GC. Given the importance of diet and dietary compounds, especially antioxidants, our results confirmed the previous findings in preventing, treating, and controlling various types of cancers. It should be noted that GC is directly related to dietary antioxidants, and the design and use of this index for this type of cancer can be an effective strategy to assess the nutritional status.

The existing relationship between dietary antioxidants with the odds of GC in this case–control study is helpful. These results are consistent with the previous studies. Serafini et al. found an association between antioxidants' equivalent and a reduction in the risk of the cardiac and non-cardiac type of GC [32]. However, Terry et al. concluded that "Antioxidant intake was not associated with the risk of gastric cardia adenocarcinoma" [35]. The impact of dietary antioxidants is mainly significant in GC-diagnosed patients under severe stress. Those who smoke, have gastric reflux, or are exposed to air pollution. According to Terry et al., intakes of the antioxidants in different types of cancers are not different, except for esophagus cancer [35]. Thus, GC etiology is diverse, and a comprehensive diet assessment can be an effective strategy for understanding its roles.

In addition, the researchers [36, 37] concluded an association between MDA and GC. They observed that medication could be used as a predictor of GC [36, 37]. In addition, this study showed that DAI as a continuous variable is associated with GC's odds. These results confirm other studies [36, 37]. Therefore, the association observed in this study between DAI, TAC, and GC indicates the importance of considering these variables' impact on nutritional assessments. So, they can even be used as predictive biomarkers of GC. Thus, this study and the previous research confirmed a strong association between the serum antioxidant status of the body and the odds of GC. Therefore, the development and validation of a suitable and non-invasive index correlated with serum antioxidants can be an option for dietary antioxidants assessment. In addition, according to the formula of DAI and considering the total energy intake of the individual, this index provides a comprehensive and universal view on the antioxidant status.

## Strengths and limitations

Using a valid FFQ is one of the strengths of this study, which provided the research by registering a complete survey of the participants' dietary intakes. Though recall bias can be a limitation of the FFQ, the proved validity and reliability of the scale, along with a trained expert administration of the survey, increase the results' confidence.

One of our study's significant strengths was to measure the serum TAC and MDA, which allowed us to investigate their association with GC and test their correlation with the designed index. This was important in interpreting the results.

However, the results of this study should be interpreted in light of some limitations. Like other case–control studies, recall bias and select bias (selecting a control group) were important challenges. However, due to the hospital-based case–control study and the use of a valid questionnaire control group's selection and the control group's selection from healthy people, these biases were minimized.

The small sample size could affect the generalization and interpretation of the results. Still, considering the study's outcome, GC, this association appears to be the same in the higher sample size studies; future studies with bigger sample sizes are recommended.

## Conclusion

The study showed that DAI is a valid indicator of dietary antioxidant assessments. It can be used as a predictor of antioxidant status due to its correlation with serum antioxidants levels. Furthermore, the results showed that dietary antioxidants have a significant relationship with GC, which indicates the importance of antioxidants in preventing this cancer. Therefore, the use of dietary antioxidants such as vitamins A, C, E, and minerals such as zinc, manganese, and selenium can be an effective strategy to prevent GC.

## Data Availability

Data will not be shared.

## Abbreviations

DAI: Dietary Antioxidant Index
GC: Gastric cancer
TNF-a: Tumor necrosis factor-a
IL: Interleukin
TAC: Total antioxidant capacity
MDA: Malondialdehyde
FRAP: Ferric-reducing antioxidant power
TBA: Thiobarbituric acid assay
FFQ: Food Frequency Questionnaire
DAQS: Dietary Antioxidant Quality Score
RDI: Recommended Daily Intake
NSAID: Nonsteroidal anti-inflammatory drug
BMI: Body Mass Index
CI: Confidence intervals
OR: Odds ratios
